# Supplementary material for: The rise of open data practices among bioscientists at the University of Edinburgh
Source: PLoS One. 2025 Jul 23;20(7):e0328065. doi: 10.1371/journal.pone.0328065 (PMC12286402; doi:10.1371/journal.pone.0328065)
Supplement: Attachment — Submitted filename: Revision.docx [file pone.0328065.s003.docx]

- **The study presents the results of original research.**

The research analyzed significantly contributes to the study of open practices in biological sciences through the evaluation of the ODDPub algorithm for assessing open data. Its originality lies in the comprehensive and longitudinal evaluation of open data practices among bioscientists within a specific institutional context, the University of Edinburgh, covering the period from 2014 to 2023. This approach adds novel insights into trends and factors driving changes in scientific data management and sharing practices.

The originality of this work is highlighted by its comprehensive and longitudinal evaluation of the evolution of open data practices among bioscientists at the University of Edinburgh, spanning a significant period (2014-2023). This specific institutional approach provides novel insights into trends and determining factors in scientific data management and sharing practices.

- **Results reported have not been published elsewhere.**

The results presented in this article are original, contributing novel findings that have not been previously reported in the literature. This work significantly enhances the understanding of open practices in biological sciences, providing new insights into the evolution of open practices.

- **Experiments, statistics, and other analyses are performed to a high technical standard and are described in sufficient detail.**

The methodology employed demonstrates a high technical standard, clearly defined through criteria of completeness, reusability, accessibility, and licensing. The complementary use of the automated ODDPub algorithm substantially strengthens the study, providing additional important validation by revealing the algorithm's sensitivity and specificity, as well as its strengths and limitations.

This section could be further enriched by incorporating additional references to previous works employing similar tools (e.g., Riedel et al., 2020 or Larsson, 2025) to clearly highlight the advantages of this hybrid methodology (manual and automated).

- **Conclusions are presented in an appropriate fashion and are supported by the data.**

The conclusions of the study are appropriate and solidly supported by the obtained data. Specific areas such as genomic data, imaging data, and human-related data are highlighted, where significant challenges remain in enhancing openness and quality in data sharing. Concrete recommendations provided could be further strengthened by citing complementary studies addressing similar challenges in other institutional or disciplinary contexts. The authors also offer practical recommendations for improving these practices.

- **The article is presented in an intelligible fashion and is written in standard English.**

The article is clearly, precisely, and academically written, with a logical and effective structure that facilitates comprehension. Effective use of figures and tables significantly enhances the presentation.

A possible improvement could be the inclusion of specific examples or additional citations about practical guidelines for writing Data Availability Statements (DAS), such as those provided by scientific journals (e.g., PLOS ONE).

- **The research meets all applicable standards for the ethics of experimentation and research integrity.**

The research fully complies with ethical norms and scientific integrity. It explicitly includes a suitable and transparent "Ethics Statement," declaring no conflicts of interest and clearly detailing funding sources.

- **The article adheres to appropriate reporting guidelines and community standards for data availability.**

The study strictly adheres to community and editorial guidelines regarding data availability and transparency. All data and codes are publicly available in recognized repositories such as Zenodo and GitHub, ensuring transparency and reproducibility.

This section could be further strengthened by explicitly mentioning adherence to FAIR principles and referencing recent publications or guidelines on these principles (Wilkinson et al., 2016).

It strictly adheres to community and editorial guidelines regarding data availability and transparency, providing complete public access to data and code through recognized repositories such as Zenodo and GitHub.

In summary, this article represents an original, significant, and rigorously documented contribution to the study of open data practices in biosciences, fully meeting all required technical, ethical, and editorial criteria. Its acceptance for publication is recommended, subject to minimal editorial modifications.

It would be beneficial to further explore the reasons behind observed differences in data sharing practices according to data types (genomic, imaging, and human). Including additional references to studies specifically addressing these technical and ethical challenges could further strengthen the discussion and comparative framework of the article (e.g., Kim, 2022 for genomic data; Williams et al., 2017 for imaging data; and Erlich & Narayanan, 2014 for human data).
