# Supplementary material for: The rise of open data practices among bioscientists at the University of Edinburgh
Source: PLoS One. 2025 Jul 23;20(7):e0328065. doi: 10.1371/journal.pone.0328065 (PMC12286402; doi:10.1371/journal.pone.0328065)
Supplement: Attachment — Submitted filename: Response to Reviewers.docx [file pone.0328065.s004.docx]

**Dear Editor and Reviewers,**

We sincerely appreciate the encouraging feedback and insightful suggestions provided by the reviewers regarding our manuscript, and the editor’s further suggestions.

We have thoroughly revised the manuscript in accordance with all the comments and suggestions from both the editor and the reviewers. Below, we detail the changes made to the manuscript. For clarity and ease of reference, we include each of the reviewers' comments and suggestions in **bold** and provide our responses subsequently in blue, highlighting modifications made in the revised manuscript with specific quotations *in* *italic*.

----------------------

\##**Editor’s Comments:**

**(not numbered, introductory text) I have missed more references that help to establish comparisons between universities, research areas and countries, both in the introduction and in the discussion section.**

Though our results do not support national-scale comparisons, we have shortened other introduction text to add examples for comparison in the new introduction paragraph below:

*A very high prevalence of data sharing has been reported in specific cases, for example over 50% of narrowly-sampled publications in 2010-2019 shared sequence of a single gene (HIV-1 pol) that is monitored for viral drug resistance (18). A study of experimental zoology reported data sharing prevalence of 19% (19), among the higher rates reported, compared to 8% in a large meta-analysis of biomedical research including the studies above (20). At the institutional level, the Charité in Berlin has developed Open Research monitoring with staff incentives, reporting data sharing rates of 7.8% in 2021 using both Open and some FAIR criteria (21).*

1) **For instance, regarding the methodology, the authors indicate that they obtain the information from the university's own database, but they do not explain how this database is covered and whether it feeds into other international or national databases. Moreover, they do not explain anything about the quality of the papers or the quality of their journals, in general, the explanation of the sample chosen is scarce.**

Thank you for this helpful comment. To clarify the nature and coverage of the university database, we have added a paragraph in the Methods section (line 108) with all relevant details about the “Research Explorer” system and the selection of articles. Note that the institutional repository at the University of Edinburgh is not at all unusual. Comprehensive institutional repositories of research articles have been the norm in UK universities for almost a decade, because of the national funding mandate.

*“Articles were retrieved from the University of Edinburgh’s public-facing research portal, Edinburgh Research Explorer (*[*https://www.research.ed.ac.uk/*](https://www.research.ed.ac.uk/)*). This portal is powered by the Elsevier Pure system and serves as a curated institutional research information system, designed to ensure the visibility, discoverability, and accessibility of all research outputs affiliated with the University of Edinburgh. This system is required for the University to fulfill our UK funder’s Open Access mandate (18). Article deposit is mandatory, and deposits and authors’ affiliations to the University are manually curated by support staff. Deposits may technically involve imports from Scopus, ORCID, PubMed and other sources but the system does not export to those external systems. The system’s coverage of research articles relevant to our analysis is very high, because the University’s core funding depends upon it.”*

And in the next paragraph, *“To avoid bias, we did not select articles by visibility or citations, though these could be explored in future work.”*

2) **In the section, Sampling Framework and Selection Process, (line 106) it is not clear if total research groups are 57 or from Medicine there are 17 research groups more.**

Thank you for pointing this out. We have clarified the description in the Methods section (line 105) as follows:

*“Of the 57 research groups included in the study, 17 were based in the College of Medicine, not in the School of Biological Sciences, but focused on similar research areas*.”

3) **Regarding the Comparison of Data Sharing Practices Across Research Areas (line 287), What is the basis (is explained in methods?) for classifying/categorizing data as genomics, imaging, human data or data warehousing (table S4)?**

Thank you for pointing out this oversight in the Methods summary, these are extensively described in our shared online methods. We have revised the Methods section (starting at line 179) to explain the classification of data types and included examples for each:

*“Data types—genomic, image, and human—were classified based on explicit mentions in the methods or results sections of each paper. Genomic data included nucleotide-based assays such as transcriptomics as well as genomic sequences but not PCR primer sequences; image data referred to primary data images suitable for reanalysis, such as microscopy; and human data included personally-identifiable information, human cell lines, or participant-derived data. For each relevant data type, we applied a scoring system: ‘Not Applicable’ (NA) if the data type was not used in the study, ‘Not Shared’ (0) if relevant data were generated but not made available, and ‘Shared’ (1) if the data were both relevant and archived in a reusable format. We also recorded the storage location of shared data, using a separate variable: ‘Repository’ for external repositories, ‘Supplementary Material’ if included in the publication’s supplementary files, ‘Both’ for cases with data in both locations, ‘DOI in Methods’ where the DOI of a previously-shared dataset, re-used in the article, was cited rather than a newly-shared dataset, and ‘NA’ where no data were shared.”*

4) **Moreover, I missed a better justification of the methods used to evaluate the level of FAIRness of the paper sample. There are different and sophisticated tools to analyze the degree of FAIR, with much more and precise items to evaluate each principle, and maybe it would be interesting to at least give an explanation why the authors chose this one and not others.**

To address this comment of editor and reviewers, we bring this issue into the Introduction:

*The methods available to assess data sharing are also changing. The manual scoring required to handle earlier, highly variable sharing practices is now supplemented by programmatic analysis of standards-compliant online infrastructure, with specialised systems to assess the 17 principles of FAIR data (14,22,23).*

We then briefly note in a new paragraph in the Methods section (line 170):

*“This approach allows for a nuanced assessment of data sharing practices, together with efficient manual scoring. FAIR guidelines did not exist in 2014 when our analysis starts, so specific evaluation of all the FAIR criteria would be most relevant in only the later years of our sample. Numerous datasets lacked sufficient metadata, for example, let alone machine-readable metadata suited to programmatic assessment. Our manual scoring system and the ODDPub software (see below) were better suited to this context and both evaluate articles, compared to more detailed and specific FAIR evaluation of datasets (see Discussion).“*

And we rearranged the Discussion to include a new paragraph that addresses several aspects of these and possible alternative methods (lines 402-404, 411-420):

*To evaluate a more scalable method, we tested the text mining algorithm ODDPub, which also scores data sharing in biomedical journal articles (23). Our study sample starts in 2014 before the FAIR guidelines existed, making current software tools that assess specifically FAIR criteria less relevant. Some software tools achieve very detailed and comprehensive assessments of FAIR metrics using machine-readable metadata, starting from a dataset URL or PID. The F-UJI tool (27), for example, assesses 16 out of 17 core FAIR metrics. Our study sample includes many datasets with no URL distinct from the parent article, and little or no metadata, let alone machine-readable metadata, making it challenging to apply such tools effectively. More importantly, approaches that start from a shared dataset cannot identify articles that shared no data, at the extreme of our Completeness criterion, or partial sharing. As ODDPub also scores articles, not datasets, we compared post hoc its detection of Open data, Open code and a DAS in the same articles to our manual assessment.*

5) **Data sharing is a highly current topic in both biomedical research and the broader landscape of science policy. It plays a central role not only in advancing knowledge, but also in shaping research evaluation and the metrics by which scientists are assessed. The authors could also discuss what policies are currently in place to address this and whether this has influenced the increased practice.**

We start a new sub-heading, “Underlying Explanatory Factors”, to address this substantial topic, as part of a major rearrangement of the Discussion. First, we address the editor’s earlier comment that more “**comparisons between universities, research areas and countries**” would be welcome, and note important caveats in making those comparisons.

*We did not directly test what motivated researchers to share their data. Survey data suggests that research data sharing was already increasing globally by 2014 (48), when our sample starts. A slightly earlier study, Roche et al. (14), indicated that 56% of ecology datasets in the Dryad repository from 2012-2013 were incomplete (Completeness score <3). Our analysis for 2014 found less sharing at the level of articles, with 77% of articles that shared any data (and could therefore have been tested by Roche et al.’s method) scoring below 3 in completeness. Assessment starting from a shared dataset clearly differs from our assessment of publications. A 2019 study on global cancer research reported that only 19% of the publications tested made some or all of their data publicly available (28). However, our 2019 sample from diverse parts of biology but including cancer among non-communicable diseases, showed that 53% of papers shared some or all of their data (completeness >2), suggesting higher data sharing. By 2023, we found pervasive data sharing in our bioscience sample, where only 8.5% of articles shared no data (Figure 2). The same manual scoring of an international sample of articles from the same year in circadian neuroscience and mental health found the converse, only 8% shared any data (31,32). Such large variations in data sharing between research domains will strongly influence institutional-level results, if the distribution of domains (or data types, as noted above) changes either over time within an institution or among institutions. Comparing institutional data, ODDPub analysis alone detected open data in 14% of articles in 2021 from the biomedical research at Charite, Berlin (21), or 8.8% of Dutch biomedical articles (49), compared to 47% of our sample from that year, or in 10.7% of articles from Linkoping University, Sweden in 2022 (50), compared to 56% of our sample that year. Even in 2014, ODDPub found 20% of our sample shared data, indicating that bioscience articles from the University of Edinburgh have higher data sharing than biomedical research elsewhere.*

We then added an expanded discussion of current policies to include global initiatives and their influence on data sharing practices (lines 491–502) :

*Beyond specific policies on research data management, scientific disciplines (54) and research institutions are increasingly considering Open Research principles in research assessment and researcher evaluation. Initiatives like DORA (Declaration on Research Assessment) (55) advocate diverse research outputs in assessment frameworks, including data sharing, while the Coalition for Advancing Research Assessment (CoARA) supports institutions to implement these policies. Proposals for DORA-compliant research assessment emphasise Open Research (56), but current academic incentives are only beginning to reflect this (57). Aligned with this broader picture, the University of Edinburgh has signed both DORA and CoARA agreements. The current, University-wide process for academic promotion does not explicitly mention any Open Research practices, so this potential incentive is unlikely to be a major factor in our results. However, a review is already underway and Open Research is one theme in broader work on Research Culture at the University, so future policy changes might reinforce the behavioral shifts revealed in our data.*

6) **Regarding the format, Table 2 needs to be improved as it currently has a poor aesthetic.**

We have revised Table 2 to ensure it aligns with the PLOS One journal formatting guidelines. ([Tables | PLOS One)](https://journals.plos.org/plosone/s/tables)

**### Journal requirements:**

1. When submitting your revision, we need you to address these additional requirements.

Please ensure that your manuscript meets PLOS ONE's style requirements, including those for file naming. The PLOS ONE style templates can be found at

<https://journals.plos.org/plosone/s/file?id=wjVg/PLOSOne_formatting_sample_main_body.pdf> and

<https://journals.plos.org/plosone/s/file?id=ba62/PLOSOne_formatting_sample_title_authors_affiliations.pdf>

2. Please note that PLOS ONE has specific guidelines on code sharing for submissions in which author-generated code underpins the findings in the manuscript. In these cases, we expect all author-generated code to be made available without restrictions upon publication of the work. Please review our guidelines at <https://journals.plos.org/plosone/s/materials-and-software-sharing#loc-sharing-code> and ensure that your code is shared in a way that follows best practice and facilitates reproducibility and reuse.
3. **We note that the grant information you provided in the ‘Funding Information’ and ‘Financial Disclosure’ sections do not match.**

**When you resubmit, please ensure that you provide the correct grant numbers for the awards you received for your study in the ‘Funding Information’ section.**

4. **Thank you for stating the following financial disclosure:**

**This project was funded by UKRI-MRC grant MR/X009726/1.**

**EW is funded by a Simons Initiative for the Developing Brain PhD studentship (SFARI #529085).**

Please state what role the funders took in the study. If the funders had no role, please state: "The funders had no role in study design, data collection and analysis, decision to publish, or preparation of the manuscript."

If this statement is not correct you must amend it as needed.

Please include this amended Role of Funder statement in your cover letter; we will change the online submission form on your behalf.

[Note: HTML markup is below. Please do not edit.]

We have revised the Funding Statement to clarify the source of funding for this project. Additionally, we have disclosed if any researchers were supported by other grants:

*“Work of HD and TZ was funded by UKRI-MRC grant MR/X009726/1 and Wellcome Trust Mental Health Award AMBIENT-BD, number Z26944/Z/23/Z, to AJM and others. MK was funded by the School of Biological Sciences, University of Edinburgh via the BioRDM team. EW was funded by the Simons Initiative for the Developing Brain PhD studentship (SFARI #529085). SC, DLU, GS, TU, and HYW did not receive specific funding for this work.”*

**###### Reviewers' comments:**

Reviewer's Responses to Questions

Comments to the Author

1. Is the manuscript technically sound, and do the data support the conclusions?

The manuscript must describe a technically sound piece of scientific research with data that supports the conclusions. Experiments must have been conducted rigorously, with appropriate controls, replication, and sample sizes. The conclusions must be drawn appropriately based on the data presented.

Reviewer #1: Yes

Reviewer #2: Yes

2. Has the statistical analysis been performed appropriately and rigorously?

Reviewer #1: Yes

Reviewer #2: Yes

3. Have the authors made all data underlying the findings in their manuscript fully available?

The PLOS Data policy requires authors to make all data underlying the findings described in their manuscript fully available without restriction, with rare exception (please refer to the Data Availability Statement in the manuscript PDF file). The data should be provided as part of the manuscript or its supporting information, or deposited to a public repository. For example, in addition to summary statistics, the data points behind means, medians and variance measures should be available. If there are restrictions on publicly sharing data—e.g. participant privacy or use of data from a third party—those must be specified.

Reviewer #1: Yes

Reviewer #2: Yes

4. Is the manuscript presented in an intelligible fashion and written in standard English?

PLOS ONE does not copyedit accepted manuscripts, so the language in submitted articles must be clear, correct, and unambiguous. Any typographical or grammatical errors should be corrected at revision, so please note any specific errors here.

Reviewer #1: Yes

Reviewer #2: Yes

**## Review Comments to the Author**

Please use the space provided to explain your answers to the questions above. You may also include additional comments for the author, including concerns about dual publication, research ethics, or publication ethics. (Please upload your review as an attachment if it exceeds 20,000 characters)

**Reviewer #1:**

• The study presents the results of original research. The research analyzed significantly contributes to the study of open practices in biological sciences through the evaluation of the ODDPub algorithm for assessing open data. Its originality lies in the comprehensive and longitudinal evaluation of open data practices among bioscientists within a specific institutional context, the University of Edinburgh, covering the period from 2014 to 2023. This approach adds novel insights into trends and factors driving changes in scientific data management and sharing practices. The originality of this work is highlighted by its comprehensive and longitudinal evaluation of the evolution of open data practices among bioscientists at the University of Edinburgh, spanning a significant period (2014-2023). This specific institutional approach provides novel insights into trends and determining factors in scientific data management and sharing practices.

• Results reported have not been published elsewhere. The results presented in this article are original, contributing novel findings that have not been previously reported in the literature. This work significantly enhances the understanding of open practices in biological sciences, providing new insights into the evolution of open practices.

1) Experiments, statistics, and other analyses are performed to a high technical standard and are described in sufficient detail. The methodology employed demonstrates a high technical standard, clearly defined through criteria of completeness, reusability, accessibility, and licensing. The complementary use of the automated ODDPub algorithm substantially strengthens the study, providing additional important validation by revealing the algorithm's sensitivity and specificity, as well as its strengths and limitations. **This section could be further enriched by incorporating additional references to previous works employing similar tools (e.g., Riedel et al., 2020 or Larsson, 2025) to clearly highlight the advantages of this hybrid methodology (manual and automated)**

We thank the reviewer for their positive assessment of our manuscript’s originality and contribution. We confirm that the results presented are original and have not been published elsewhere, as preprint sharing is permitted in PLoSone guidelines.

We appreciate the reviewer’s recognition of the technical quality of our methodology and the complementary role of ODDPub. We have already cited *Riedel et al. (2020)*, which introduced the ODDPub tool, and clarified our hybrid manual-automated approach further in the Methods section (line 229):

“*This hybrid approach—integrating manual evaluation with automated scoring—provides a deeper validation of ODDPub’s performance and highlights the evolving nature of data documentation.”*

And we summarise in Discussion:

*Where data-sharing rates are low, however, ODDPub analysis can quickly identify at least a significant fraction of the few articles with shared data (33), and its developers have described just such a workflow at institutional level (21).*

If we have identified the correct Larsson 2025, then this report on ODDPub implementation in a Swedish University (10.3384/report-119790) is also now cited as (50) in the revised Discussion, which is quoted in full above in response to the editor’s comment 4):

*Comparing institutional data, ODDPub analysis alone detected open data … in 10.7% of articles from Linkoping University, Sweden in 2022 (50), compared to 56% of our sample that year.*

2) Conclusions are presented in an appropriate fashion and are supported by the data. The conclusions of the study are appropriate and solidly supported by the obtained data. Specific areas such as genomic data, imaging data, and human-related data are highlighted, where significant challenges remain in enhancing openness and quality in data sharing. **Concrete recommendations provided could be further strengthened by citing complementary studies addressing similar challenges in other institutional or disciplinary contexts.** The authors also offer practical recommendations for improving these practices.

We appreciate the reviewer’s comments. This issue has been addressed in response to the editor’s comment #6 and reviewer comment #5 (below). In the new Discussion sections, we discussed relevant disciplinary and institutional policies and challenges related to openness. Specific comparisons to other contexts are also provided in the Discussion (lines 455–476), with relevant citations.

3) The article is presented in an intelligible fashion and is written in standard English. The article is clearly, precisely, and academically written, with a logical and effective structure that facilitates comprehension. Effective use of figures and tables significantly enhances the presentation. **A possible improvement could be the inclusion of specific examples or additional citations about practical guidelines for writing Data Availability Statements (DAS), such as those provided by scientific journals (e.g., PLOS ONE).**

Thank you for the feedback regarding the clarity and structure of the manuscript. We clarified the point in the Introduction (line 60):

*“Journals such as PLOS ONE and Nature have mandated Data Availability Statements for all research articles, requiring authors to clearly specify how and where their data can be accessed”*

The suggestion on practical guidelines is out of the scope of this article, which focuses on analysis rather than guidance. We have recently published guidance elsewhere, see refs. 32 and 38.

The research meets all applicable standards for the ethics of experimentation and research integrity. The research fully complies with ethical norms and scientific integrity. It explicitly includes a suitable and transparent "Ethics Statement," declaring no conflicts of interest and clearly detailing funding sources.

4) The article adheres to appropriate reporting guidelines and community standards for data availability. The study strictly adheres to community and editorial guidelines regarding data availability and transparency. All data and codes are publicly available in recognized repositories such as Zenodo and GitHub, ensuring transparency and reproducibility. **This section could be further strengthened by explicitly mentioning adherence to FAIR principles and referencing recent publications or guidelines on these principles (Wilkinson et al., 2016).**

In response to the suggestion to explicitly mention the FAIR principles, we have added the following in the Methods section (line 245):

“The data and code have been shared in accordance with the FAIR principles (6).”

5) It strictly adheres to community and editorial guidelines regarding data availability and transparency, providing complete public access to data and code through recognized repositories such as Zenodo and GitHub. In summary, this article represents an original, significant, and rigorously documented contribution to the study of open data practices in biosciences, fully meeting all required technical, ethical, and editorial criteria. Its acceptance for publication is recommended, subject to minimal editorial modifications. **It would be beneficial to further explore the reasons behind observed differences in data sharing practices according to data types (genomic, imaging, and human). Including additional references to studies specifically addressing these technical and ethical challenges could further strengthen the discussion and comparative framework of the article (e.g., Kim, 2022 for genomic data; Williams et al., 2017 for imaging data; and Erlich & Narayanan, 2014 for human data).**

This point is addressed in the Discussion, under new subheading “*Variation among data types*”, where we analyse differences in openness across data types. We now cite complementary studies as suggested:

- “This aligns with observations by Kuo et al (38), who noted that the presence of well-established repositories and community norms significantly facilitates data sharing in genomics.” (line 433)
- “ These challenges are further compounded by issues such as the complexity involved in sharing multi-modal imaging data and the high cost of acquiring accurate data labels, as highlighted by Du et al (41).” - (line 439)

**Reviewer #2:**

1) The theoretical framework is appropriate, as it explains the privacy or confidentially concers in a complex field such as bioscience research. However, what is missing is a more precise delimitation of what is the frontier of knowledge in this field, i.e., what is known and what is not known about the subject under analysis, **why is it important and what are the benefits of sharing data, concerns, legislation or mandates and challenges?**

It would also be desirable to have a more detailed description of what are the characteristics and complexity of data in this field, what type of data predominate, in what formats, what difficulties can be encountered in its management and deposition, etc.

We thank the reviewer for this insightful comment, which was addressed in response to Reviewer 1’s comment #5. Specifically, we expanded the Discussion section under new subheading “*Variation among data types*” to include comparisons across data types and noted broader technical and ethical challenges. Additionally, we discussed relevant international and national data policies under new subheading “*Underlying explanatory factors*”.

2) In the Discussion section, **it is necessary to debate the advantages and disadvantages of the assessment system used by the authors of the manuscript with respect to the use of automated tools already developed and tested,** such as F-UJI - Automated FAIR Data Assessment Tool, developed by the FAIRsFAIR project (<https://www.f-uji.net/>).

This point has been thoroughly addressed in response to Editor’s comment #5 and Reviewer 1’s comment #1. We compare our manual scoring approach with automated tools, now specifically citing F-UJI, and explain our rationale for selecting a manual system with a comparison to ODDPub. After a summary in the Methods section, the relevant text is lines 411-428 in Discussion.

3) **They should discuss the importance of awareness training programs** in data management for researchers, especially in the field of knowledge addressed.

We agree with the reviewer about the importance of training in data management. In response, we added a dedicated paragraph to the Discussion and cited relevant literature: (line 483)

*“Training programs have been identified as effective methods to foster open research practices and data sharing among researchers (35,40). Our team provided local, bioscience-specific training for researchers from 2021 (35) that became mandatory for bioscience PhD students from 2022, in addition to broader data management training from the University and growing external provision.”*

**Discuss whether or not the management, storage and curation of data should be a task entrusted to researchers, technical support staff or information science specialists, as well as the advantages and disadvantages of each of these options.**

Though we agree that personnel and expertise for data management is a critical issue, the results that we present have no bearing on this question, so we suggest that weighing the benefits of three possible types of staff is out of scope for this article.

Our team has directly contributed to data management in the domain of interest but not to any of the articles scored here. Moreover, the articles were selected by different team members, who were blind to any such contribution.

6. PLOS authors have the option to publish the peer review history of their article (what does this mean?). If published, this will include your full peer review and any attached files.

If you choose “no”, your identity will remain anonymous but your review may still be made public.

Do you want your identity to be public for this peer review? For information about this choice, including consent withdrawal, please see our Privacy Policy.

Reviewer #1: No

Reviewer #2: No

**----------------------------------------------**

We would like to thank the reviewers and the editor once again for their constructive feedback and thoughtful suggestions, which have helped us improve the clarity, depth, and rigor of our manuscript. We believe the revisions made have strengthened the paper considerably, and we hope it now meets the expectations for publication in your journal.

Please do not hesitate to contact us if further clarification or revision is required.
Sincerely,
